# Bacterial Distribution in the Rhizosphere of Wild Barley under Contrasting Microclimates

**DOI:** 10.1371/journal.pone.0017968

**Published:** 2011-03-23

**Authors:** Salme Timmusk, Viiu Paalme, Tomas Pavlicek, Jonas Bergquist, Ameraswar Vangala, Triin Danilas, Eviatar Nevo

**Affiliations:** 1 Department of Forest Mycology and Pathology, Uppsala BioCenter, Swedish University of Agricultural Sciences (SLU), Uppsala, Sweden; 2 Department of Gene Technology, Tallinn University of Technology, Tallinn, Estonia; 3 Institute of Evolution, University of Haifa, Mt. Carmel, Haifa, Israel; 4 Department of Analytical and Physical Chemistry, Uppsala University, Uppsala, Sweden; 5 Institute of Forestry and Rural Engineering, Estonian University of Life Sciences, Tartu, Estonia; Auburn University, United States of America

## Abstract

**Background:**

All plants in nature harbor a diverse community of rhizosphere bacteria which can affect the plant growth. Our samples are isolated from the rhizosphere of wild barley *Hordeum spontaneum* at the Evolution Canyon (‘EC’), Israel. The bacteria which have been living in close relationship with the plant root under the stressful conditions over millennia are likely to have developed strategies to alleviate plant stress.

**Methodology/Principal Findings:**

We studied distribution of culturable bacteria in the rhizosphere of *H. spontaneum* and characterized the bacterial 1-aminocyclopropane-1-carboxylate deaminase (ACCd) production, biofilm production, phosphorus solubilization and halophilic behavior. We have shown that the *H. spontaneum* rhizosphere at the stressful South Facing Slope (SFS) harbors significantly higher population of ACCd producing biofilm forming phosphorus solubilizing osmotic stress tolerant bacteria.

**Conclusions/Significance:**

The long-lived natural laboratory ‘EC’ facilitates the generation of theoretical testable and predictable models of biodiversity and genome evolution on the area of plant microbe interactions. It is likely that the bacteria isolated at the stressful SFS offer new opportunities for the biotechnological applications in our agro-ecological systems.

## Introduction

Bacteria and other microorganisms contribute greatly to the Earth's biomass as they form the bottom of the food chain and orchestrate the cycling of carbon, nitrogen, and flow of other nutrients through the ecosystem. They are the ‘dark matter’ of life and may also hold the key to various global problems facing our society e.g. generating sources of nutrition and energy, developing powerful new pharmaceuticals, and cleaning up the environmental disorder. To date, there are a limited number of microbial species that have been studied in the laboratory. The most well-known of these are perhaps *E. coli* and *B. subtilis*. However even their wild relatives differ substantially from the highly subcultured laboratory representatives.

**Figure 1:**
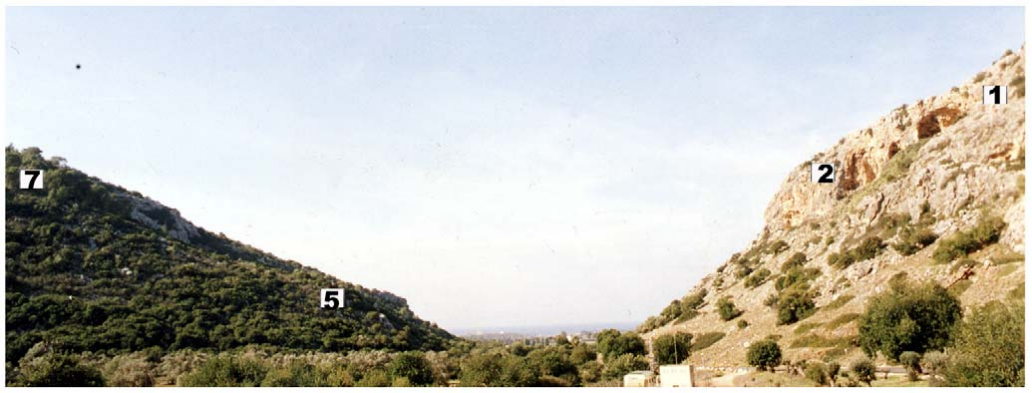
Cross section of the ‘Evolution Canyon’ indicating the collection sites on South Facing Slope (SFS) 1 and 2 and North Facing Slope (NFS) 5 and 7.

In the study reported in this manuscript, samples were collected from the ecological laboratory called Evolution Canyon (EC) which is found in northern Israel (Fig.1). The ‘African’ or south-facing slopes (AS or SFS) in canyons north of the equator receive higher solar radiation than on the adjacent ‘European’ or north-facing slopes (ES or NFS). This difference in solar radiation is associated with higher maximal and average temperatures and evapotranspirations on the more stressful ‘African’ slope. It causes dramatic physical and biotic interslope divergence, which may have originated several million years ago after mountain uplifts [1]. These canyons are extraordinary, natural, evolutionary laboratories. Rocks, soils, and topography are similar on the opposite slopes (50–100 m apart at the bottom); microclimate remains the major interslope divergent factor. So far the intraspecific interslope divergence has been compared in 2500 species across various life forms from prokaryotes through eukaryotic lower and higher plants, fungi, and animals [2], [3], [4] unraveling the link between environmental stress and genome evolution in adaptation. This unique ecological situation facilitates the generation of theoretical testable and predictable models of biodiversity and genome evolution.

Soils are heterogeneous environments with various dynamic parameters in which any of the parameters can affect microbial growth and survival. Soil is generally nutrient poor; its content of organic matter typically varies in concentration from 0.8 to 2.0%. Hence, native soil bacteria constantly face nutrient deprivation. The root surface and the region immediately surrounding a root, constitutes an ecological niche in soil where nutrients (root exudates) are more readily available. Certain bacteria have developed mechanisms to take advantage of this niche. The root exudates are released as organic material from the roots as they grow through the soil. The process of root exudation enhances microbial growth and also contributes to the structure of microbial communities in the rhizosphere. Root exudates contain a significant fraction of a plant's photosynthate, estimated to be ∼20% of the carbon allocated to the root system [5]. The exudates contain a variety of compounds, such as amino acids, simple sugars and other organic acids that are passively released from the roots. There are also actively secreted compounds such as carbohydrates and enzymes; mucilage (sloughed-off cells and cell lysates); and gases, such as carbon dioxide and ethylene. The bacteria that typically populate the area around the root (rhizobacteria) can affect plant growth in various ways. Thus, they may increase plant growth and/or reduce susceptibility to diseases caused by various pathogenic agents [6]. In addition to facilitating biotic stress alleviation, a number of reports have been published regarding the activity of some plant growth-promoting bacteria to confer upon plants the ability to tolerate abiotic stresses such as drought, salt and nutrient deprivation [7], [8], [9], [10]. Here, we report a comparison of the rhizobacterial relationship with wild barley roots under stressful SFS and moderate NFS conditions.

Ethylene one of the gaseous components in plant root exudates is known as a plant stress hormone. It was first shown by Glick and collaborators that bacteria containing ACC deaminase (ACCd) can cleave ACC which is an immediate precursor of ethylene synthesized in plant tissues [11]. Certain plant growth promoting rhizobacteria enhance plant stress tolerance through 1-aminocyclopropane-1-carboxylate deaminase (ACCd) and provide significant protection to a wide range of plant species from the damage caused by various stress conditions such as flooding, metals, temperature extremes, the presence of organic environmental contaminants and high salt [12], [13], [14], [15]. Hence, the ACCd bacteria act as sink for ACC and lower the level of inhibitory ethylene.

Here, we report a study of the distribution of culturable bacteria from contrasting microclimates as reflected by the organisms found in the rhizospheres of wild barley, *Hordeum spontaneum*, plants [16]. It is well known that only a very small fraction of the soil microbial community can be cultured. Culture-independent molecular techniques are usually applied to investigate microbial community structure and microbial diversity in a variety of environments. The purpuse of this study was merely to find bacteria with new opportunities for the biotechnological applications in agricultural systems. We have characterized the bacteria based on their ACCd content, biofilm forming properties, phosphorus solubilization activity and halotolerance. To the best to our knowledge, this is the first study where the bacterial traits are correlated with plant growth in the environment under varying stress conditions. The features are likely to have provided a selective advantage for the plant-bacterial biofilm complex survival, and the bacteria may have helped the plant to tolerate various stresses using one or more of those mechanisms. The bacteria co-evolved with their hosts, over millennia, are likely to control, to large extent, plant adaptation to the environment and have potentially huge applications to our agro-ecological systems enhancing plant stress tolerance.

## Materials and Methods

### Rhizosphere sampling and sample preparation

The sampling of wild barley rhizospheres was performed as described previously (Timmusk 2009). Briefly, five wild barley plants were collected from each of the SFS (SFS1 and SFS2) and NFS (NFS5 and NFS7) sun and shaded stations at ‘EC’ in April, two weeks before maturation. The SFS has stations 1, 2, 3 and they are at 120, 90, and 60 meters above sea level. Three stations on the NFS (5, 6 and 7) are 60, 90, and 120 meters above sea level respectively (Fig. 1). The plant roots were carefully shaken and washed in sterile distilled water to remove all loosely attached soil and to collect bacteria intimately linked to the plant root. Plants were placed in new plastic bags, transferred to the laboratory, and then stored at +4°C until they were processed the next day.

Plant rhizosphere material (1 g) was homogenized as described by the manufacturer using FastPrep Instrument (BIO 101). Hence, the rhizosphere macerate contains bacteria in the wild barley endorhizophere, very close to the rhizoplane. The bulk soil samples were also collected from the sites described above, and 1 g of the material was homogenized as described above.

SFS1 and SFS2 samples as well NFS5 and NFS7 samples were pooled resulting in SFS sun and shadow plant rhizosphere and bulk soil as well as NFS sun and shadow plant rhizosphere and bulk soil material. Bulk soil and plant rhizosphere material was suspended in sterile PBS (137 mM NaCl, 2.7 mM KCl, 10 mM sodium phosphate dibasic, 2 mM potassium phosphate monobasic, pH of 7.4).

The content of endospore-forming bacteria was determined after heat treatment of the soil or plant material suspension at 80°C for 30 min. Tryptic Soy Agar (TSA) plates were inoculated with 100 µL of these suspensions, corresponding to 10^−3^–10^−5^ g soil or plant rhizosphere material per plate. All agar media contained 15 g agar and 50 mg cycloheximide, to reduce fungal growth, and had a pH of 7. The inoculated petri dishes were incubated for several weeks at, room temperature (∼21°C), 30°C, 37°C, or 40°C in boxes together with a beaker of water (to prevent drying of the agar).

For spore detection soil and plant rhizosphere material was Shaeffer-Fulton stained [17]. Spore concentration was detected using a bright line hemacytometer counting chamber (Model 3900, Hausser Scientific Company, Horsdam, PA, (USA).

### Screening for bacterial metabolic properties

Tiny amounts of 60 colonies from the biomass was picked from the TSA plates and transferred to Tryptic Soy Broth (TSB) medium for a biofilm production assay. The remainder of each colony was streaked on to

1. Dworkin and Foster (DF) plates for the ACC deaminase assay [18];
2. NBRIP medium [19] for the P solubilization assay;
3. high salt medium for halophilcity assays.

Finally, the TSA plates were used to ensure that some of each colony remained. The colonies for the endospore forming bacteria were identified by 16SDNA sequencing from that plate.

### Assays

#### Biofilm formation

The bacteria were picked from TSA plates, resuspended in TSB liquid medium and diluted to a final optical density at 600 nm (OD_600_) of 0.02. Cultures were transferred to standing culture vessels. Polysterone 96 well microtitre plates were filled with 150 µl of culture per well. The cultures were allowed to stand at 30°C for the specified time. The extent of biofilm formation was assayed by staining with crystal violet. After the incubation period, the cultures were removed, and microtitre plates were gently washed three times with 150 µl of sterile water to remove loosely associated bacteria, then dried at 30°C for 30 min. Samples were stained by the addition of 1% crystal violet solution to each well to initial inoculation level and incubated for 20 min. The vessels were then washed. The intensity of crystal violet staining was measured after the addition of dimethyl sulfoxide to each dry well. The samples were incubated for 20 min, after which the OD_590_ values were measured on a plate reader. All samples were tested in seven independent wells. The colonies with OD_590_ values over 1 were considered as good biofilm formers.

#### ACC deaminase production

Bacteria were screened for the ability to use ACC as a sole nitrogen source by streaking onto DF plates as described by Penrose and Glick [18]. Plates were incubated for 48 h at either 25°C or 30°C§.

#### Phosphorus solubilization

The ability of bacteria to solubilize phosphorus was determined using NBRIP medium [19]. The plates were incubated at 30°C for 7 days and then assessed for colony diameter and solubilization zone diameter (halo zone). Solubilization index was evaluated according to the ratio of the total diameter of a colony with its halo zone and the colony diameter [20]. Colonies with an index higher than 2 were considered good phosphorus solubilizers. Change of pH by the endospore forming bacteria in broth cultures was determined by pH meter after seven days of incubation.

#### Halophility assay

For halophility assays, bacteria were transferred to a medium containing the following ingredients (per liter). 5.0 g casamino acids (Difco), 5.0 g yeast extract (Difco), 1.0 g sodium glutamate·H_2_O, 3.0 g trisodium citrate·2H_2_O, 2.0 g KCl, 0.2 g MgSO_4_·7H_2_O, 36 mg FeCl_2_·4H_2_O, 116 g (2 M) NaCl and 20 g Bacto-agar (Difco), pH 7.2, followed by incubation at 37°C for 2 weeks.

### Bacterial identification

DNA was isolated from1-day-old cultures on agar plates. Single colonies were resuspended to obtain a bacterial density of about10^5^ cfu ml^−1^. A 0.3 µl aliquot of the bacterial culture was suspended with 4.7 µl of buffer (10 mM Tris-HCl pH 7.6; 50 mM KCl, 0.1% Tween 20). For lysing, the suspension was heated and immediately cooled on ice. The mixture was centrifuged at 6000 *g* for 5 min and the supernatant was used for PCR. Aliquots of 10 µM of primers1492R (5′-GGTTACCTTGTTACGACTT-3′) and 27F (5′-AGAGTTTGATCCTGGCTCAG-3′) and 1 µl of template were used. The reaction was performed in 10 µl. The reactions conditions were 95°C for 2 min followed by 30 cycles of denaturation at 95°C for 15 s, annealing at 55°C 20 s, primer extension at 72°C for 1 min, followed by the final extension at 72°C for 5 min. For sequencing the PCR products were purified with QIAquick™ Gel Extraction kit (QIAGEN, Hilden, Germany).

### Statistics

Phosphorus solubilization data were submitted to ANOVA and Fishers least significant difference test (P≤0.05) using the Minitab statistical package (Minitab Inc. State College PA). To test the significance of the interslope differences, we also applied the “Interslope Difference” (ID) model based on the Median test [21]. According to this model, the interslope difference is significant (P≤0.05) if the estimates of the chosen parameter are higher on at least three independent samples on one slope than the estimates of the same parameter on the same number independent samples of the opposite slope. This model is rather conservative but eliminates some collection biases.

## Results

The SFS and NFS sun and shaded area culturable bacterial numbers were estimated on TSA plates (Table 1). Samples were taken from plant rhizospheres (surface bacteria + endophytes; see Material and Methods) and bulk soil. As expected, the bacterial populations in the SFS as well as the NFS plant rhizospheres were significantly higher (10^6^–10^7^) than the populations in bulk soil (∼10^4^) (ID model, P<0.05).

**Table 1:** Bacterial distribution on the ‘EC’SFS and NFS slopes.

		EC South Facing Slope				EC North Facing Slope		
	S		Sh		S		Sh	
	Plant material1	Bulk soil1	Plant material	Bulk soil	Plant material	Bulk soil	Plant material	Bulk soil
Total bacterial count^1^	10^6^±0.4×10^6^	10^4^±0.2×10^4^	10^6^±0.4×10^6^	10^4^±0.3×10^4^	10^6^±0.3×10^6^	10^4^±0.5×10^4^	10^7^±0.2×10^7^	10^4^±0.2×10^4^
Spore ratio to vegetative cells^2^ (%)	0.5	55	0.6	60	0.7	7	0.5	12
ACC utilization^3^ (%)	50	1	52	1	5	1	3	1
Biofilm formation^4^ (%)	55	2	50	1	4	1	5	1
Halophilic growth^5^ (%)	51	1	55	1	5	1	4	1
P solubilization^6^ (%)	50	2	49	2	3	1	2	2

1 The data are expressed per gram of fresh weight. Wild barley roots were prepared as described in Material and Methods.

Each data point represents three independent experiments. The culturable aerobic fraction of the total bacterial CFUs was determined on TSA plates as described in Material and Methods s-sun area; sh-shaded area.

2 Spores were counted using Shaeffer-Fulton staining.

3 ACC (1-Aminocyclopropane-1-carboxylate) medium was composed of salts for *P. polymyxa* minimal medium there the nitrogen source is replaced with ACC.

4 Biofilm formation was estimated as described in Material and Methods. The isolates with OD >1 were considered as biofilm formers.

5 Halophilic growth was determined in the medium containing 2 M NaCl (see Material and Methods).

6 P solubilization was estimated using NPRJP medium (see Material and Methods). The colonies with index >2 were considered as P solubilizers.

We also estimated spore to vegetative cell ratios at SFS and NFS. These ratios are relatively low in both slope's plant rhizospheres (0.5–0.6 and 0.5–0.7 respectively)–an expected result since the rhizosphere is the area for nutritional deposition where fresh nutrition is available for vegetative cells. On the other hand, the ratio differs considerably between SFS and NFS bulk soil (55–60% and 7 to 12%) reflecting the more stressful conditions at the SFS slope (ID model, P<0.05) (Table 1). For reproducibility, all the bacterial platings were performed three times.

### Bacterial metabolic properties

Sixty colonies from the SFS and NFS sun and shaded area plates (corresponding to 10^−3^–10^−4^ g soil or plant rhizosphere material per plate) were screened for: 1) ACC deaminase production, 2) biofilm formation, 3) phosphorus solubilization and 4) halophilic growth.

To estimate the number of ACCd containing bacteria, tiny amounts of the colony were streaked on to the defined medium ([22] there the nitrogen source was replaced with ACC, hence the ACC was a as a sole nitrogen source). A significantly higher number of bacteria from SFS (both from sun and shaded areas) grew on ACC plates (50 and 52% respectively) in comparison to bacteria from the NFS (5 and 3% respectively) (Table 1). This likely reflects the major role of the enzyme to help plants to withstand stress; in this case, in the wild barley rhizosphere at SFS. This conclusion is supported by the fact that bulk soil from both slopes contains relatively low number of ACCd bacteria (∼1%) (ID model, P<0.05) (Table 1).

A tiny amount of bacteria of the same colony from the TSA plate was streaked on to high salt medium plates (2 M NaCl medium corresponding to a moderately halophilic bacterial medium) [23]. The colonies from the SFS performed much better on the high salt medium (51 to 55% grew, respectively). From the NFS, only 4 to 5% of colonies could grow on the high salt medium. In the bulk soil, the number of bacteria able to grow on the high salt medium was significantly smaller at ∼1% (ID model, P<0.05).

NBRIP plates were used to screen for the ability of bacterial isolates to solubilize phosphorus. Bacteria from the SFS sun and shaded area rhizospheres displayed the greatest ability to solubilize P with a solubilization index of 49–50%. According to the ID model the corresponding numbers were relatively low at NFS sun and shaded area rhizospheres (2–3%) and even lower in bulk soil of both slopes (1–2%) (P<0.05) (Table 1).

These results indicate a significantly higher level of ACC utilization, biofilm formation, phosphorus solubilization and moderately halophilic bacteria from the SFS wild barley rhizosphere in comparison to rhizosphere samples from the NFS (Table 1). Sun and shaded area did not make much difference in any of the measured characteristics (Table1).

All of the reported data was highly reproducible and all assays were repeated three times.

### Endospore forming bacterial identification

As described earlier, SFS 1 sun and shadow, SFS2 sun and shadow, NFS5 sun and shadow, and NFS7 sun and shadow samples were pooled and treated at 80°C for 30 min. TSA plates were inoculated with 100 µL of these suspensions, corresponding to 10^−3^ g soil or plant rhizosphere material per plate. A total of 41 bacterial isolates from the NFS and 35 isolates from the SFS were identified by 16S rDNA sequencing. Among the SFS isolates, 10 were identified as *Bacillus megaterium*, 15 as *P. polymyxa*, 5 as *B. cereus* and 5 as *B. pumilus*. The same species were identified from the NFS TSA plate with 15, 1, 10, and 6 isolates, respectively (BLAST homology 98–100%).

### Endospore forming bacterial metabolic properties

**Figure 2:**
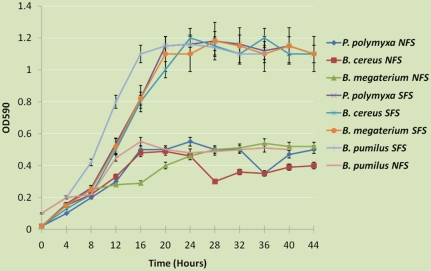
Solid surface assay of the South Facing Slope (SFS) and North Facing Slope (NFS) bacterial biofilm formation. The crystal violet assay was used to measure solid surface biofilm formation at 30C. Preparation and analysis were as described in Material and Methods.

The bacterial isolates were screened for ACCd activity, biofilm formation, halophility and phosphorus solubilization. All *B. megaterium* isolates at SFS contained ACCd, were good biofilm formers, were able to grow on the high salt medium, and eight were good phosphorus solubilizers (Fig. 2 and Table 2). At the same time none of the NFS slope *B. megaterium* population showed any of these metabolic activities. It was similar for *P. polymyxa* metabolic activity at SFS (Fig 2 and Table 2). We could identify only one *P. polymyxa* isolate at NFS. This correlates well with our previous results where SFS was reported to have a significantly higher *P. polymyxa* population [24]. The NFS *P. polymyxa* isolate was not able to utilize ACC, was not a significant biofilm producer and neither grew on halophilic medium nor showed P solubilization (Fig 2 and Table 2). Most of the *B. cereus* and *B. pumilus* SFS isolates could utilize ACC, were biofilm formers, moderately halophilic and showed phosphorus solubilization activity (Fig 2 Table 2 and Table 3). Phosphorus solubilization activity correlated well with pH change in the medium (Table 3). Additionally, using tandem mass spectrometry we identified acid phosphatase in the bacterial culture filtrates from SFS (Bergquist unpublished data). At the same time, none of the NFS *B. cereus* neither *B. pumilus* isolates had the reported activities (Fig 2 Table 2 and Table 3). These results support the results shown on Table 1. Surprisingly, when the SFS isolates were stored for three weeks at −80°C and then rescreened for their halophilic ability, the isolates were only able to grow at 1.3 M NaCl.

**Table 2:** Endospore forming bacterial distribution on the ‘EC’ SFS and NFS slopes.

	‘EC’ South Facing Slope					‘EC’ North Facing Slope				
	No of isolates^1^	ACC utilization^2^	Biofilm formation^3^	Halophilic growth^4^	P. soulbilization^5^	No of isolates	ACC utilization	Biofilm formation	Halophilic growth	P.solubi-lization
*P. polymyxa*	15	15	10	8	1	1	0	0	0	0
*B. megaterium*	10	10	10	10	8	15	0	0	0	0
*B. cereus*	5	5	5	5	3	10	0	0	0	0
*B. pumilus*	5	5	5	5	4	6	0	0	0	0

1 The endospore forming bacterial fraction was isolated as described in Material and Methods.

2 ACC (1-Aminocyclopropane-1-carboxylate) medium was composed of salts for *P. polymyxa* minimal medium there the nitrogen source is replaced with ACC.

3 Biofilm formation was estimated as described in Material and Methods. The isolates with OD >1 were considered as biofilm formers.

4 Halophilic growth was determined in the medium containing 2 M NaCl (see Material and Methods).

5 P solubilization was estimated using NBRIP medium (see Material and Methods). The colonies with index >2 were considered as P solubilizers.

**Table 3:** Endospore forming bacterial phosphorus solubilization.

Isolate^1^	Solubilization index^2^	pH change^3^
*P. polymyxa SFS* (15)	3±0.5*a	4.1±0.5a
*P. polymyxa NFS* (1)	1.5±0.3b	5.5±0.4b
*B. megaterium SFS* (10)	4±0.3a	4.2±0.4a
*B. megaterium NFS* (15)	1±0.2b	5.6±0.6b
*B. cereus SFS* (5)	4.5±0.5a	4.0±0.5a
*B. cereus NFS* (10)	1.5±0.3b	5.7±0.5b
*B. pumilus SFS* (5)	3.5±0.4a	4.3±0.4a
*B. pumilus NFS* (6)	0.5±0.2b	5.8±0.5b

1 Endospore forming bacterial isolates as shown in Table 2. Solubilization index and pH change was studied of each isolate in three independent experiments.

2 Bacterial phosphorus solubilization index was determined after bacterial growth in NBRIP agar plates for 7 days at 30C (See Material and Methods).

3 Change of pH by the bacteria was determined after 7 days of incubation in NBRIP broth (See Material and Methods).

*P. polymyxa, B. megaterium, B. cereus and *B. pumilus* SFS and NFS isolate values (mean value±SE) were compared. Numbers followed by different letter are significantly different (P<0.05) according to Fisher's least significance difference test.

## Discussion

Maintenance of homeostasis is pivotal to all forms of life. Since plants cannot escape environmental fluctuations, their sensitive mechanisms must be evolved to allow the rapid perception of stress and recognize the pattern for adaptation and survival. Re-establishment of homeostasis in response to environmental stress requires reprogramming of metabolism and gene expression to move energy sources from growth related biosynthetic processes to defense acclimation and ultimately adaptation. Failure to do so can result in irreversible senescence and death. Microbial biofilms formed in the rhizosphere of wild barley have coevolved with the plant over long period of time [16]. SFS biofilms differ from the NFS biofilms and it was suggested that the pattern of biofilm formation reflects a tight mutual dependence between plant and rhizobacteria at the stress and non-stress environment [16]. Rhizobacteria can affect plants in various ways [6]. Besides facilitating biotic stress alleviation also abiotic stress tolerance has been reported earlier [7], [8], [9], [10]. We have inoculated *Arabidopsis thaliana* and Swedish wheat cultivar with *P. polymyxa* strains from SFS and NFS. SFS isolate inoculations resulted in enhanced drought tolerance while NFS strains did not (manuscrpt in preparation).

Enhancemet of plant osmotic stress tolerance is a very complex process involving various networks from both plant and rhizobacterial side. Despite the complexity of the networks, the plant's response must be versatile because of changing ecological and environmental pressures. How do we interpret the striking difference in the SFS and NFS rhizobacterial physiological characteristics? It is well known that under stress conditions the plant hormone ethylene endogenously regulates plant homeostasis and reduces plant growth. Degradation of the ethylene precursor ACC by bacterial ACCd decreases plant stress so that plant growth can continue. The results presented here indicate that the number of bacteria that have ACCd is significantly higher in the rhizosphere samples of the South Facing Slope than in samples from the North Facing Slope of the Evolution Canyon in northern Israel. At the same time the numbers of bacteria do not differ in the bulk soil (Tables 1 and 2). It is likely that the stress that exists on SFS (compared to NFS) causes higher ethylene and ACC levels around the roots. Hence it is expected that the bacterial numbers able to utilize ACC as a nitrogen source will be higher in the rhizosphere of stressed plants.

It is well known that bacteria attach to roots, and various mechanisms are known for attachment including the involvement of a variety of cell components such as outer membrane proteins, wall polysaccharides (capsules), lipopolysaccharides, and cell surface agglutinin. In addition, exopolysaccharide is produced by bacteria in the rhizosphere. This ability not only provides many advantages to bacterial cells, it also enhances soil aggregation, which in turn improves water stability, which is critical to the survival of the plant. Hence, there is a strong selective advantage for the production of a slimy layer of extracellular polymeric substances in the rhizosphere especially under the SFS stressful conditions. Thus, biofilm forming bacteria may protect plants using the protective biofilm layer by mechanisms such as niche exclusion [25], [26], [27]. This ability of bacteria to protect both themselves and their plant hosts may be synergistically strengthened by inducing the basic protection of enhancing homeostasis via ACC breakdown [12], [14], [28], [29].

The experiments reported here also show that the bacteria at the SFS side (both sun and shade) are significantly better P solubilizers than bacteria from the NFS side and that the activity was much higher in the plant rhizosphere than in bulk soil. It was also observed that while all of the slime producers were relatively good biofilm producers, they were not P solubilizers (Table 1 and 2). The change is pH correlated well with the P solubilizing activity (Table 3). Thus, in agreement with others [30], organic acids are probably directly involved in the formation of soluble P. The NFS and SFS soils are classified as Terra Rossa according to the soil classification system [31]. Both slopes have shallow eroded profiles, clay-like in texture and with reddish B horizons of prismatic or block-like structures, dark colored A horizons and abundant stones. However the SFS soils show signs of greater erosion and exposure to limestone blocks and karren. Hence, the P solubilization ability provides a selective advantage for the bacterial plant association at that slope. We speculate that ability to produce extracellular polymeric substances may contribute by maintaining the bacterial population in close contact with the insoluble mineral during the process of chelation.

The property of halophilism is widespread in bacteria; several bacterial strains have been used to improve plant salt stress tolerance [32]. Bacterial halophiles are abundant in environments such as salt lakes, saline soils. The isolates from the stressful SFS had a remarkably higher tolerance to salt stress than did the bacteria from the NFS, notwithstanding the fact that the AC and ‘EC’ slopes do not differ in their salinity levels. However, a similar phenomenon was found in the soil fungus *Aspergillus niger*. The SFS population has a tendency to be more adaptively resistant than the European to stress associated with low water activity [33]. While fresh bacterial isolates could grow well on 2 M salt, the phenomenon was not stable and the bacteria from the storage cultures were only able to grow on up to 1.3 M salt. How do we unravel the bacterial halophilic behavior? Drought and heat stress often provokes similar responses to salt stress, i.e. osmotic stress. Organisms adapt to that stress by accumulating osmolytes to keep the intercellular ionic concentrations at low levels maintain turgor pressure, and cell volume as well as by changing the properties of their cytoplasmatic membrane. The types of organic molecules used for osmotic balance include polyols, sugars amino acids, betaines, and ectoines and occasionally peptides suitably altered to remove charges. Osmolytes can either be synthesized by the cell or transported into the cell from the medium. A key feature of these molecules is that they do not inhibit overall cellular functioning, although they may modulate individual enzyme activities. From the perspective of plant survival, it is possible that osmolytes produced by the bacteria were taken up by plant cells and used to adapt to stress at SFS. Additionally, the polysaccharide production that was used for the slime envelope could play a role in supporting bacterial survival in the high salt environment.

It is often suggested that microbial diversity is a result of habitat heterogeneity [34]. However, the culturable endospore forming bacteria isolated from the two climatically contrasting slopes belong to the same taxonomic units (Table 2). On the other hand, despite the prevalence of the same species, the bacteria differ in ACCd, biofilm production, phosphorus solubilization and osmotic tolerance (Table 2). It is generally accepted that bacteria through various mechanisms can acquire genetic information from the surrounding environment. Moreover, recombination frequencies and mutation rates tend to increase under stressful conditions [35], [36], [37]. Rates of evolutionary change may therefore be enhanced in adverse environments. Endospore forming bacteria may remain dormant for long periods and germinate in conditions that are favorable to growth. Their survival strategies, in addition to acquiring new genetic information, involve multilayered cell wall structures; formation of stress resistant endospores; and secretion of peptide antibiotics, peptide signal molecules and enzymes. These stress ‘survival’ strategies also suggest that the bacteria might have been present over a long period of time in the rhizosphere of the plant which is a progenitor of modern barley. However one thing is clear, it is the interaction of the hormones that control growth, development, and reproduction, as well as a plant's response to environmental stress. A change in one group automatically switches in changes in the other groups.

In other studies it has been shown that ACCd can modulate a complicated mechanism of plant growth regulation based on the regulation of IAA and ethylene levels [38] and references therein). It has been suggested that ACCd may have originated from genes arisen by convergent evolution following modification and duplication of bacterial genes encoding pyridoxal phosphate-requiring amino acid deaminases or aminotransferases ([38] and references therein). Soil bacteria may also have acquired the genes by horizontal gene transfer ([38] and references therein). As mentioned above, these genes can regulate plant stress under various biotic and abiotic stress conditions. Taken together, it is possible that this enzyme might be an early signaling molecule mediating basic stress tolerance at SFS and other stressful environments. Hence, future studies need to be directed towards isolating and characterizing the ACCd gene and its complex regulatory region[39] from the SFS isolates.

In conclusion, we have shown that the stressful SFS slope contains significantly higher population of ACCd containing, biofilm forming, phosphorus solubilizing, osmotic stress tolerant bacteria. Our results are in agreement with Kolter and Greenberg ([40] and references therein) that the bacteria on the plant root behave much like a multicellular organism. They excrete the ‘matrix’ to provide a buffer against the environment and hold themselves in place. Hence, whatever is produced inside the biofilm has a suitable environment and higher probability to get through to the target. We suggest that the rhizosphere bacteria, together with the plant roots at the SFS wild barley rhizosphere, might function as communities with elevated complexity and plasticity which, in aggregate, have afforded the plant the adaptability to the harsh conditions encountered over millennia. The features discussed above are likely to have provided a selective advantage for the plant-bacterial biofilm complex survival and the bacteria may have helped the plant to tolerate various stresses using one or more of those mechanisms. How do the different organisms interact in the complex? For a start, bacteria have systems that monitor and respond to quorum sensing signals from the same as well from other species. Nevertheless, much remains to be discovered regarding the complex nature of the *H. spontaneum*-microbial interaction.
